# FAM117A and PIGU regulate the trilogy of gastric carcinogenesis

**DOI:** 10.1016/j.csbj.2025.08.036

**Published:** 2025-09-07

**Authors:** Nianzhi Chen, Yueqiang Wen, Qingsong Liu, Jing Du, Dan Yao, Maoyuan Zhao, Cui Guo, Tingyao Wang, Jia Ma, Jianyuan Tang, Yumei Wang, Jinhao Zeng

**Affiliations:** a TCM Prevention and Treatment of Metabolic and Chronic Diseases Key Laboratory of Sichuan Province, Hospital of Chengdu University of Traditional Chinese Medicine, Chengdu 610072, China; b Shuguang Hospital Affiliated to Shanghai University of Traditional Chinese Medicine, Shanghai 201203, China; c School of Basic Medical Sciences, Chengdu University of Traditional Chinese Medicine, Chengdu 611137, China; d School of Clinical Medicine, Chengdu University of Traditional Chinese Medicine, Chengdu 611137, China; e State Key Laboratory of Ultrasound in Medicine and Engineering, College of Biomedical Engineering, Chongqing Medical University, Chongqing 400016, China; f School of Chinese Materia Medica, Beijing University of Chinese Medicine, Beijing 100029, China

**Keywords:** Gastric carcinogenesis, FAM117A, PIGU, CAM, Cell proliferation

## Abstract

Gastric cancer (GC) develops through a multistep process; however, the molecular mechanisms driving the progression from normal gastric mucosa to precancerous lesions and ultimately to GC remain incompletely understood. Here, this study performed whole-transcriptome sequencing and ATAC-seq on normal gastric mucosa (N), gastric precancerous lesions (P), and gastric tumor tissues (T) to profile chromatin accessibility, lncRNAs, miRNAs, and mRNAs. Differential expression and KEGG pathway analyses identified key hub genes driving gastric carcinogenesis. A core competing endogenous RNA (ceRNA) network revealed critical regulatory lncRNAs for H19 and SNHG3. Through integrative analysis of ATAC-seq and whole-transcriptome sequencing, FAM117A and PIGU were identified as potential key regulatory factors. Experimental validation, including western blot, RT-qPCR, plasmid transfection and immunohistochemistry, confirmed FAM117A and PIGU as pivotal targets. Functional assays demonstrated that FAM117A and PIGU regulate the p53 signaling pathway and modulate cell adhesion molecules (N-cadherin and E-cadherin), highlighting their involvement in abnormal proliferation and tumor metastasis during GC progression. These findings identify FAM117A and PIGU as novel biomarkers and potential therapeutic targets, providing mechanistic insights into gastric carcinogenesis.

## Highlights

• FAM117A and PIGU regulate the trilogy of gastric carcinogenesis.
• Cell adhesion molecules and abnormal cell proliferation are key mechanisms in the development of gastric cancer.
• FAM117A and PIGU regulate the p53 signaling pathway and the expression of N-cadherin and E-cadherin.

## Graphical Abstract

**Figure:**
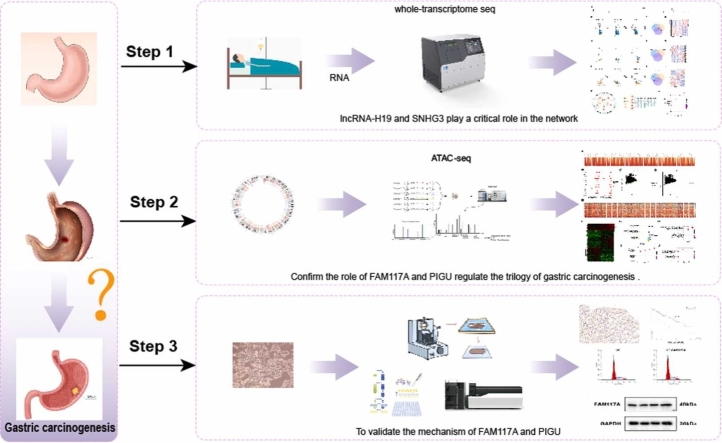

## 1 Introduction

Gastric cancer (GC) is one of the most common cancers in the world. The incidence rate of gastric cancer ranks fifth, and the mortality rate ranks third among common cancers globally, with increasing incidence and mortality in China [1]. According to the Correa cascade model, the development of gastric cancer in humans is a multi-step process [2], [3]. Chronic atrophic gastritis (CAG), intestinal metaplasia (IM), and dysplasia form the "trilogy" of gastric carcinogenesis. These lesions are collectively referred to as gastric precancerous lesions (GPLs)[4], which are at the highest risk of progressing to early gastric cancer (EGC) [5]. This trilogy delineates the complete progression from normal gastric mucosa to precancerous lesions, culminating in gastric cancer. Therefore, it is crucial to explore strategies to inhibit the progression from GPL to EGC through prevention and treatment. Additionally, elucidating the molecular targets underlying GPLs is essential. Unveiling the authentic molecular mechanisms representing early neoplastic stages, such as GPLs, and understanding the transition from normal gastric mucosa to premalignant lesions and to EGC, are of paramount importance to improve the cure rate of EGC. However, despite extensive research on the molecular mechanisms of GC, specific molecular biomarkers for predicting the transition in the trilogy of gastric carcinogenesis remain unclear.

Recent studies have highlighted that a large number of non-coding RNAs (ncRNAs) are involved in regulating gene expression through various mechanisms. Their dysregulation is associated with the pathogenesis of GC and can be utilized clinically as diagnostic and prognostic biomarkers [6], [7], [8]. miRNAs bind to the 3 '-untranslated region (UTR) of messenger RNA (mRNA) to inhibit translation [9]. Conversely, lncRNAs can inhibit miRNAs and promote translation by acting as sponges or competitive endogenous RNAs (ceRNAs) [10], [11]. Numerous studies have demonstrated that miRNAs and lncRNAs are implicated in key cellular processes of gastric cancer, including differentiation, proliferation, apoptosis, migration, invasion, and metastasis of gastric cancer cells [12], [13]. Many miRNAs are differentially expressed in GC patients and can be used to identify people with increased susceptibility to GC [14], [15]. Moreover, miRNAs regulate the expression of numerous genes and pathways, and alterations in their expression may activate oncogenes or silence tumor suppressors, including the phosphoinositol 3-kinase/Akt/mammalian target of rapamycin (PI3K/AKT/mTOR), mitogen-activated protein kinase (MAPK), and Wnt/β-catenin pathways [16], [17], [18], [19], [20]. In addition, lncRNA-MT1JP has emerged as a potential therapeutic target by competitively binding to miRNAs in gastric cancer and regulating FBXW7 expression, and has emerged as an important epigenetic regulator that plays a key role in GC [21]. These ncRNAs can act as ceRNAs to influence the expression levels of target genes, ultimately functioning as tumor promoters in GC. The ceRNA mechanism represents a regulatory network in which ncRNAs naturally and competitively bind to miRNAs, thereby inhibiting miRNA-mediated regulation of target genes, such as lncRNAs, and forming a complex miRNA-mediated ceRNA network [22]. Although the ceRNA network in GC has been reported in various studies, its mechanisms in the entire trilogy of gastric carcinogenesis have been less explored. Therefore, elucidating the role of ceRNAs in the trilogy of gastric carcinogenesis holds significant biological and clinical importance.

Currently, assay for transposase-accessible chromatin with high-throughput sequencing (ATAC-seq) is a next-generation technology for deciphering epigenetic maps and systematically detecting genome-wide open chromatin regions based on Tn5 transposable enzyme activity that can be used to predict molecular targets for disease [23], [24]. At the same time, full transcriptome sequencing methods can also be used to study many different aspects of RNA biology [25]. The combination of these two approaches allows for more comprehensive identification of molecular biomarkers, and more accurate identification of EGC target genes and molecular mechanisms.

To identify potential novel biomarkers for EGC and GPLs, and to elucidate the molecular mechanisms underlying the transition from normal gastric mucosa to GPLs and EGC, we performed whole-transcriptome sequencing and ATAC-seq analysis on gastric tissues from 30 patients. These tissues included tumor tissues (T) from patients with early gastric cancer, gastric mucosa (P) from patients with precancerous lesions, and normal gastric mucosa (N) from healthy subjects. Differential expression analysis was conducted between the T and P groups, and between the N and T groups, to identify key regulatory genes. Upstream transcriptional regulatory factors were further analyzed using ATAC-seq. Subsequently, we performed functional interaction predictive analysis to study gene regulatory circuits. Through these analyses, we identified FAM117A and PIGU as potential regulators in the trilogy of gastric carcinogenesis, supported by data from The Cancer Genome Atlas (TCGA) and in vitro experiments. Overall, our findings provide new insights into the mechanisms of premalignant tissue transformation into EGC, offering a basis for early gastric cancer diagnosis and suggesting potential therapeutic targets for GPLs and EGC.

## 2 Materials and methods

### 2.1 Participants and sample collection

A total of 30 samples were collected (10 gastric cancer tissues (T group) from patients who underwent operative treatment, 10 gastric precancerous lesion tissues (P group) and 10 normal gastric mucous membrane tissues (N group) from mucosal biopsy specimens from the gastric tract) by gastroscopy (N group: 5 males and 5 females; P group: 6 males and 4 females; T group: 6 males and 4 females; age<60: N 8 people; P 5 people; T 4 people; age≥60: N 2 people; P 5 people; T 6 people) at the Hospital of Chengdu University of Traditional Chinese Medicine, Chengdu, Sichuan, between 2018.12–2020.9 from patients with no preoperative radiation or chemotherapy or drug treatment. All specimens were snap frozen in liquid nitrogen and stored at -80 °C. This study was approved by the Ethics Committee of the Hospital of Chengdu University of Traditional Chinese Medicine (approval no. 2018KL‑023), and informed consent was obtained from all participants.

### 2.2 RNA extraction and quality control

Total RNA was extracted from tissue of three groups by using TRIzol reagent (Life Technologies CA, USA) following the manufacturer’s instructions. RNA degradation and contamination were monitored on 1.5 % agarose gels. RNA concentration and purity were measured using a NanoDrop 2000 Spectrophotometer (Thermo Fisher Scientific, Wilmington, DE). RNA integrity was assessed by using the RNA Nano 6000 Assay Kit for the Agilent Bioanalyzer 2100 System (Agilent Technologies, CA, USA).

### 2.3 Bioinformatics analysis

To ensure data consistency and comparability, clustering analysis was performed on the sequencing samples to identify and exclude outlier samples with abnormal expression profiles. Ultimately, samples with relatively consistent gene expression patterns were selected for each group (N = 7, P = 6, T = 7). Pairwise differential expression analyses among the three groups (N vs. P, N vs. T, and P vs. T) were conducted using the R package DESeq2. Significantly differentially expressed mRNAs, lncRNAs, and miRNAs were identified based on predefined thresholds (e.g., P < 0.05 and fold change ≥ 1.5). Based on the commonly differentially expressed lncRNAs, miRNAs, and mRNAs, a ceRNA regulatory network (lncRNA–miRNA–mRNA) was constructed by integrating information from established miRNA target prediction databases, including miRDB, TargetScan, and miRTarBase. The ceRNA network was visualized using Cytoscape v3.10.0. KEGG pathway enrichment analysis was performed on the differentially expressed mRNAs within the ceRNA network using the R package clusterProfiler, aiming to identify key biological pathways involved in gastric cancer progression. Pathways with both P-values and false discovery rate (FDR) < 0.05 were considered significantly enriched.

### 2.4 Cell lines

Human Gastric Epithelial Cells (GES-1) were provided by Beyotime (Shanghai, China). AGS, HGC27, MKN45 cells were purchased from pricella (Wuhan, China). The medium supporting GES-1, AGS, HGC27 and MKN45 cells growth was RPMI 1640 medium (Gibco) added with 10 % fetal bovine serum (FBS, PAN Biotech, Germany) plus 1 % penicillin-streptomycin (Gibco). Cells were maintained in a 37 °C cell incubator containing 5 % CO_2_.

### 2.5 Real time quantitative polymerase chain reaction (RT-qPCR) analysis

GES-1, AGS, HGC27, MKN45 cells (6 × 10^^5^/well) were seeded into 6-well plate and cultured overnight. Total RNAs were extracted from cells using UNlQ-10 column total RNA purification kit (Sangon Biotech, Shanghai, China). And then cDNAs of samples were obtained after RNAs of cells were reversed transcription using SureScriptTM first-strand cDNA synthesis kit (GeneCopoeia, Guangzhou, China). A 7500 Real Time PCR System (Applied Biosystems, USA) was used to RT-qPCR analysis according to the guide of BlazeTaqTM SYBR Green qPCR Mix 2.0 (GeneCopoeia, Guangzhou, China). The relative mRNA expression levels were quantified using the 2 ^-ΔΔCT^ method, and were normalized to GAPDH. The involved primer sequences are shown in Table 3.

### 2.6 Histopathology and immunohistochemistry (IHC) analysis

Firstly, the gastric mucosa tissues obtained from patients and normal people were fixed, embedded and sliced with 4 % paraformaldehyde. After antigen repair, the slices were cleaned with washing buffer, and each slice was closed with sealing solution at room temperature for 1 h. The blocking solution was removed and the antibody antigen was cross-linked with the sample using FAM117A and PIGU antibodies. Finally, microscope was used to observe and record the staining of samples.

### 2.7 Western blotting analysis

Cells were lysed with RIPA (Tris-HCl: 50 mM, NaCl: 150 mM, 1 % deoxycholate, 1 % Triton-100) buffer containing 1 % protease inhibitor (TargetMol, Shanghai, China). The total protein of cellular was obtained by centrifugation at 1,2000 g and 4°C for 10 min. 10 % SDS-PAGE gel was used to isolate the protein samples, and then wet transfer was carried out. After the transfer, 5 % skim milk powder was closed at room temperature for one hour. The protein was incubated with the primary antibody for overnights at 4°C, and the film was washed with Tris-buffered saline–Tween 0.1 % (TBS-T) at least three times for five minutes each time, and then the HRP-conjugated secondary antibody GAPDH (Proteintech, 1:5000) was incubated in a room temperature shaker for hours. The film was washed repeatedly with TBS-T, and finally the protein image was obtained by chemiluminescence reagent (4 A Biotech, Beijing, China) combined with a protein exposure instrument.

### 2.8 Transfection

For the overexpression of FIM117A or knockout of PIGU, AGS cells were seeded at a density of 1 × 10⁶ cells per well in 6-well plates and cultured for 24 h. Subsequently, the cells were transfected with FIM117A plasmid (GenePharma, China) or siPIGU (GenePharma, China), or Vector (GenePharma, China) using Lipo6000™ reagent (Beyotime, Shanghai, China) according to the manufacturer's instructions. The transfection mixture was prepared in DMEM medium without PBS and applied to the cells for 6 h. After transfection, the medium was replaced with fresh complete culture medium, and the cells were incubated for an additional 24 h. Each experiment was performed in triplicate and repeated three times to ensure reproducibility.

### 2.9 Cell viability assay

AGS cells were seeded into a 96-well plate at a density of 5 × 10 ³ cells per well and cultured overnight in DMEM medium supplemented with 10 % fetal bovine serum (FBS) and 1 % penicillin/streptomycin (P/S). Following overnight culture, the cells were transfected with either the OE-FIM117A plasmid or SI-PIGU according to the manufacturer's protocol. Cell viability was measured using a Cell Counting Kit-8 (CCK-8) (Beyotime, Shanghai, China) after 24, 48, and 72 h of culture, respectively. Each experiment was performed in quadruplicate and repeated three times to ensure reproducibility.

### 2.10 Cell cycle analysis

After transfection of AGS cells, the population stage of cells was detected using cell cycle and apoptosis analysis kit (Beyotime, Shanghai, China) according to instruction.

### 2.11 Statistical analysis

All experimental data are showed as mean ± SEM. Statistical difference analysis was determined by Student’s t test between two groups, and one-way ANOVA when groups were at least three. P < 0.05 was considered statistically significant.

## 3 Results

### 3.1 H19 and SNHG3 were identified as the key lncRNAs, and the cell cycle and p53 signaling pathways were the critical drivers in the progression of gastric cancer

**Fig. 1:**
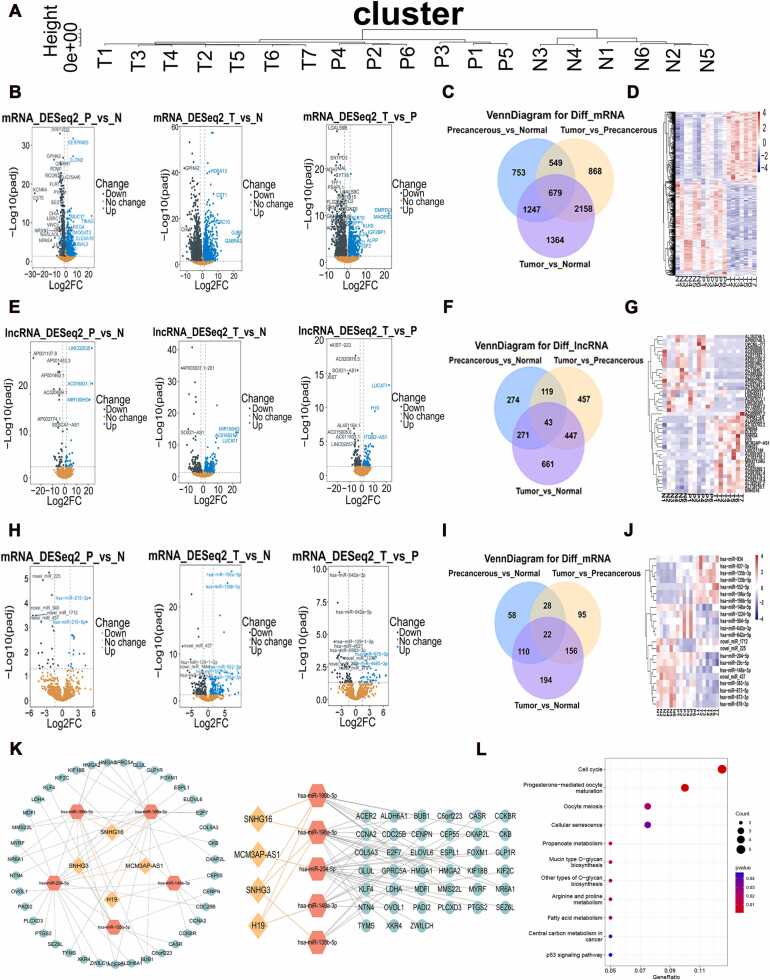
Constructed a ceRNA network by the DEGs from the N, P and T groups. (A) Transcriptome resequencing of normal gastric mucosa (N, n = 7), precancerous lesions (P, n = 6), and gastric cancer tissues (T, n = 7). (B) Differential mRNA analysis (DEGs) of N and P, N and T, P and T. (C-D) The three groups of DEGs were cross-picked. (E) Differential lncRNA analysis of N and P, N and T, P and T. (F-G) The three groups of diff-lncRNAs were cross-picked. (H) Differential miRNA analysis of N and P, N and T, P and T. (I-J) The three groups of different miRNAs were cross-picked. (K) The ceRNA network. (L) KEGG enrichment analysis of DEGs in ceRNA network. count: the number of genes that are enriched in each KEGG pathway from the input gene list.

In this study, we investigated the key regulatory genes involved in the progression of gastric cancer (GC) by analyzing the transcriptomic profiles of gastric mucosal tissues from three groups of patients: normal gastric mucosa (N group), gastric precancerous lesions (P group), and gastric cancer tumor tissues (T group). Full transcriptome sequencing was performed, encompassing mRNA, miRNA, and lncRNA profiling. As shown in Fig. 1A, following outlier classification of the samples obtained through sequencing, we selected samples with similar gene expression profiles from each group (N = 7, P = 6, and T = 7). Differential expression analysis of mRNA between the N and P, N and T, and P and T groups was conducted to identify key regulatory genes involved in the progression from normal mucosa to precancerous lesions and to cancer (Fig. 1B). Interestingly, 679 mRNAs, 43 lncRNAs, and 22 miRNAs were identified as overlapping differentially expressed genes (DEGs) across the three groups (Fig. 1C-J). These RNAs may serve as crucial regulatory elements in the development of GC. To further explore the interactions among these DEGs, a competing endogenous RNA (ceRNA) network was constructed (Fig. 1K). In this ceRNA network, the number of direct connections a node has with other nodes (node degree) reflects its interaction potential. Higher-degree nodes typically represent key regulatory hubs. Our ceRNA network analysis revealed that both H19 and SNHG3 directly interacted with three miRNA nodes each. Notably, miR-196a-5p and miR-204a-5p exhibited particularly dense connections with their respective mRNA targets. Based on these connectivity patterns, we identified H19 and SNHG3 as the most important lncRNAs and miR-196a-5p and miR-204a-5p as the most critical miRNAs in the network. KEGG pathway enrichment analysis of the DEGs revealed that the cell cycle and p53 signaling pathways were the main drivers of gastric cancer progression (Fig. 1L).

### 3.2 PBX3 and ZNF263 were the main transcription factors regulating this process through ATAC-seq analysis

**Fig. 2:**
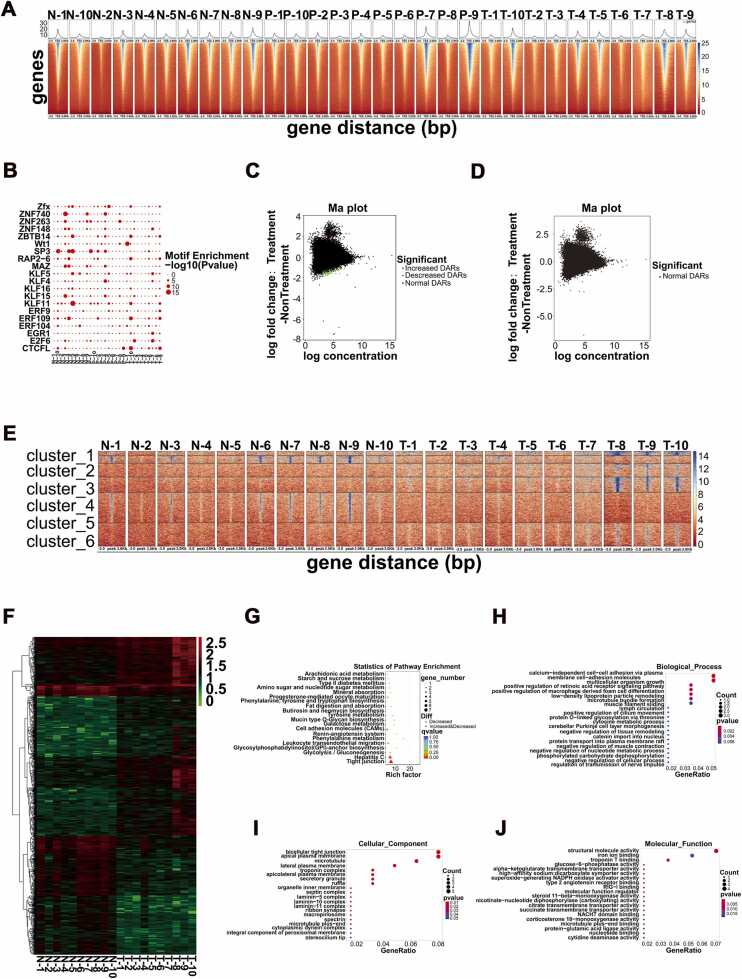
ATAC-seq analysis of differential peaks revealed PBX3 and ZNF263 as upstream transcription factors. (A) ATAC-seq analysis of gastric mucosa tissues from N, P, and T groups (n = 10). (B) Enrichment diagram between motifs of all samples. (C) The DARs of N vs T. (D) The DARs of P vs T. (E) ATAC-seq differential peaks heatmap (k-means clustered, deepTools)**.** (F) The heat map obtained after standardization with the count value of N vs T differential peak in each sample. (G) KEGG enrichment analysis of DARs associated gene. (H-J) Go enrichment analysis of DARs associated gene.

After analyzing the core regulatory target genes, we conducted ATAC-seq analysis on gastric mucosal tissues from patients in the N, P, and T groups (n = 10) to further investigate the upstream transcription factors. Using deepTools version 3.2.0, we generated read density distribution heatmaps spanning ±3 kb around the transcription start site (TSS) of each gene. The results demonstrated a significant enrichment of reads around the TSS in all samples, confirming that the experiment successfully captured the authentic open chromatin states (Fig. 2A). Next, we extracted and identified the peaks for each gene group. The identified peak regions, which represent open chromatin regions containing transcription factor binding sites (TFBS), play a crucial role in regulating transcription factor-mediated gene expression. We then identified the representative motif sequences enriched in these peak regions. The enrichment of these motifs, compared to known transcription factors in the database, is shown in Fig. 2B. We observed that the ZNF and KLF gene families were most frequently enriched. Subsequently, we analyzed the peak differences (DARs) between the N vs. T and P vs. T groups. Significant DARs were observed between the normal group and the gastric cancer group, while no significant DARs were detected between the GPL and GC groups (Fig. 2C and D). Therefore, the DARs obtained from the N vs. T comparison were selected for further analysis to identify key transcriptional regulatory factors involved in the development of gastric cancer (GC). The ATAC-seq read enrichment heatmap (Fig. 2E) and the peak difference heatmap (Fig. 2F), both generated using the deepTools toolkit, confirmed that the DARs between the N and T groups were significantly different. In this study, genes associated with these DARs were further enriched and analyzed. KEGG enrichment analysis (Fig. 2G) and GO enrichment analysis (Fig. 2H-J) revealed that tight junctions and cell adhesion molecules (CAMs) were the most critical factors in the progression of GC. The detected motif sequences were compared with known motifs using Tomtom, as shown in Table 1. PBX3 and ZNF263 were identified as the main enriched motifs.

**Table 1:** ATAC-seq analysis identified differential core upstream transcription factors

No	**factor**	**p-value**	**Overlap**	**Query consensus**	**Target consensus**
1	PBX3	0.0005	16	TGCCTGTAATCCCAGCACTTTGGGAGGC	CCGCCTGTCACTCACCC
2	Plagl1	1.13283e−05	13	GAGGCGGGAGAATCGCCTGGGCCCAGGAGG	CCCTGGGGCCAGG
3	RAV1(var.2)	0.00031	12	GAGGCGGGAGAATCGCCTGGGCCCAGGAGG	ATCACCTGAGGC
4	ZNF263	0.00034	12	GAGGCGGGAGAATCGCCTGGGCCCAGGAGG	GGGGGGAGGAGG

### 3.3 FAM117A and PIGU are newly discovered regulated prognostic regulatory genes

**Fig. 3:**
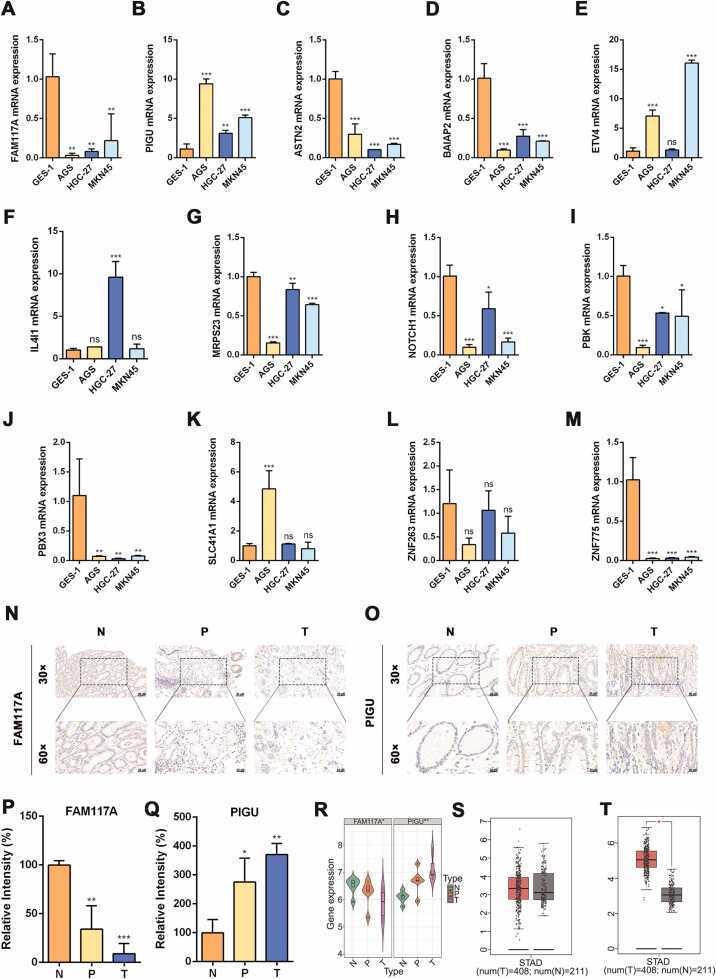
FAM117A and PIGU are prognostic genes regulating gastric cancer. (A-M) The relative mRNA expression of FAM117A, PIGU, ASTN2, BAIAP2, ETV4, IL4I1, MRPS23, NOTCH1, PBK, PBX3, SLC41A1, ZNF263 and ZNF775 in GES-1, AGS, HGC-27 and MKN45 cells. (N-O) The representative IHC images of FAM117A and PIGU in gastric mucosal tissues of groups N, P, and T, and quantitative results shown in (P-Q). Scale bar = 50 µm. (R) The transcriptome level of FAM117A and PIGU in gastric mucosal tissues of group N, group P and group T. (S-T) The contents of FAM117A and PIGU in normal group and gastric cancer group were analyzed on the website of http://gepia.cancer-pku.cn. (*P＜0.1, **P＜0.01, ***P＜0.001. n = 3).

DEGs associated with ZNF263 and PBX3, as identified from the database, are presented in Table 1. Several genes with potentially strong associations were selected in Table 2 for subsequent validation. To assess expression differences between normal gastric mucosa and gastric cancer cells, GES-1, AGS, HGC-27, and MKN45 cell lines were used, the primers were showed in Table 3. RT-qPCR results indicated that, compared with GES-1 cells, the expression levels of FAM117A, ASTN2, BAIAP2, MRPS23, NOTCH1, PBK, PBX3, and ZNF775 were significantly downregulated in AGS, HGC-27, and MKN45 cells. Among these, FAM117A, PBX3, and ZNF775 exhibited the most pronounced decreases. In contrast, PIGU expression was significantly upregulated in AGS, HGC-27, and MKN45 cells, while EVT4 expression was elevated in AGS and MKN45 cells. IL4I1 expression was significantly increased only in HGC-27 cells, and SLC41A1 expression was significantly elevated only in AGS cells. No significant differences were observed in ZNF263 expression (Fig. 3A-M). FAM117A and PIGU expression was further examined in human tissue specimens via immunohistochemistry. FAM117A expression was markedly decreased in tumor (T) and precancerous (P) tissues compared with normal (N) tissues, whereas PIGU expression was elevated in the T and P groups relative to the N group (Fig. 3N-Q). Additionally, transcriptome sequencing of mRNA revealed that the expression trends of FAM117A and PIGU as DEGs were consistent with these observations, showing gradual downregulation and upregulation, respectively, across the three groups (Fig. 3R). These expression patterns were further validated using the GEPIA (Gene Expression Profiling Interactive Analysis) database (http://gepia.cancer-pku.cn), confirming higher FAM117A expression in the N group relative to the T group (Fig. 3S) and higher PIGU expression in the T group relative to the N group (Fig. 3T).

**Table 2:** The expression profiles of DEGs strongly correlated with PBX3 and ZNF263 across different cancer types

**DEGs**	**Target_genes**	PBX3|Average	**NSCLC**	**lymphoma**	**CRC**	**Neuroblastoma**	**Hepatoma**
ASTN2	ASTN2	1075.6	1218	1932	0	2228	0
BAIAP2	BAIAP2	1072	1226	1985	0	2149	0
SLC41A1	SLC41A1	998.2	865	1882	0	2244	0
FUT8	FUT8	923.6	802	1740	0	2076	0
DGLUCY	DGLUCY	881	863	1665	0	1877	0
IL4I1	IL4I1	747.8	625	1356	0	1758	0
FAM117A	FAM117A	697.4	341	1286	0	1860	0
ZNF775	ZNF775	635.6	251	1246	0	1681	0
MAGI1	MAGI1	579.8	990	197	0	1712	0
NOTCH1	NOTCH1	344.6	423	651	0	649	0
ZBTB7C	ZBTB7C	272.4	0	506	0	856	0
MAGI3	MAGI3	267.6	0	396	0	942	0
HOXC9	HOXC9	210.8	354	181	0	519	0
FBXL6	FBXL6	207.4	147	417	0	473	0
MRPS23	MRPS23	153.8	238	0	0	531	0
IRX3	IRX3	145.6	0	0	0	728	0
CASZ1	CASZ1	118.4	0	0	0	592	0
SEC14L1	SEC14L1	112	81	0	0	376	103
ABTB2	ABTB2	111.8	102	209	0	248	0
TRIB3	TRIB3	109.8	183	202	0	164	0
PCDH9	PCDH9	107.2	233	0	0	189	114
TAF4	TAF4	104	225	0	0	295	0

**Symbol**	**Target_genes**	ZNF263|Average	293 T	**Hepatoma**	**Leukemia**	**Leukemia**
FAM117A	FAM117A	879.428571	1718	1746	351	216
FUT8	FUT8	778.857143	2175	1692	0	0
PIGU	PIGU	581.285714	1593	1346	0	0
MRPS23	MRPS23	580.714286	1359	919	190	284
ETV4	ETV4	474.142857	1018	1207	0	0
PBK	PBK	467.428571	1875	636	0	0
BAIAP2	BAIAP2	408.428571	1060	478	172	207
ITPR1	ITPR1	389.571429	1815	407	0	0
IRX3	IRX3	387.428571	1315	807	0	0
CDC14A	CDC14A	344.285714	1469	485	0	0
ARHGAP10	ARHGAP10	277.571429	866	772	0	0
CLDN3	CLDN3	264.857143	1333	190	0	0
PAPSS1	PAPSS1	196.857143	609	334	0	0
ARHGAP24	ARHGAP24	193.285714	398	482	0	0
EPHA5	EPHA5	155	953	132	0	0
BMPR1B	BMPR1B	149.857143	1049	0	0	0
NOTCH1	NOTCH1	147.285714	783	142	0	0
TRIB3	TRIB3	144.714286	180	434	0	0
SEC14L1	SEC14L1	139.571429	373	335	0	0
TTLL6	TTLL6	136.714286	957	0	0	0
ZBTB7C	ZBTB7C	133.571429	741	73	0	0
CD9	CD9	129.285714	795	0	0	0
CLDN7	CLDN7	128.428571	899	0	0	0
ABTB2	ABTB2	118.714286	488	167	0	0
DGLUCY	DGLUCY	116.571429	548	182	0	0
THSD4	THSD4	116.571429	816	0	0	0
SLC41A1	SLC41A1	110.571429	507	177	0	0
CA9	CA9	108	120	303	0	0
ADAMTS13	ADAMTS13	106	300	139	0	0
NFIC	NFIC	100.857143	494	0	0	0

**Table 3:** The primer sequences for strongly correlated DEGs

Gene name	Primer sequences(5′-3′)
Human FAM117A	Forward:CATTCTCCCTGGACACCATCCT Reverse:CACCTTCTAGCTCTTGCCAGGA
Human PIGU	Forward:GCCATCACACTGACCTTCAACG Reverse:TGTGCCATCCTTGGCGGTCAAG
Human ASTN2	Forward:TACGATGCCACTTCGAGCACCA Reverse:GGAGTCAGGTTTGAGGCACTTG
Human BAIAP2L2	Forward:CGAGGTCTACTTCAGTGCCATC Reverse:TCCAGGTCAGAGTTCAAGTGCC
Human ETV4	Forward:AGGAACAGACGGACTTCGCCTA Reverse:CTGGGAATGGTCGCAGAGGTTT
Human IL4I1	Forward:CTACCAGATGGCTCTCAACCAG Reverse:GACATCACGTCTCCCAGAAGCT
Human MRPS23	Forward:TAAGACGAGTAGGCGAAGCAAGG Reverse:TCTTCCAACGCAGTCTGTGGTC
Human NOTCH1	Forward:GGTGAACTGCTCTGAGGAGATC Reverse:GGATTGCAGTCGTCCACGTTGA
Human PBK	Forward:GGAGTCTCTCTACCACTGGATG Reverse:CACCATTCTCCTCCACAGCTTC
Human PBX3	Forward:CCAGTGAAGAAGCCAAAGAGGAG Reverse:CAGCATAGAGGTTGGCTTCTTCC
Human SLC41A1	Forward:TGGCTGTCTTCACGCCTGTGAT Reverse:GAGCTTGCTCAGAGTTCTCTCC
Human ZNF263	Forward:AGGTGGGACAAGGAGGAAAGCT Reverse:GCATACAGACGGAACACCTTCC
Human ZNF776	Forward:TGGAGTCTCCTTAGTGAGGCTC Reverse:GGTCTGCTTAGAAGGTGTCTCG

### 3.4 DARs and DEGs conduct joint analysis

**Fig. 4:**
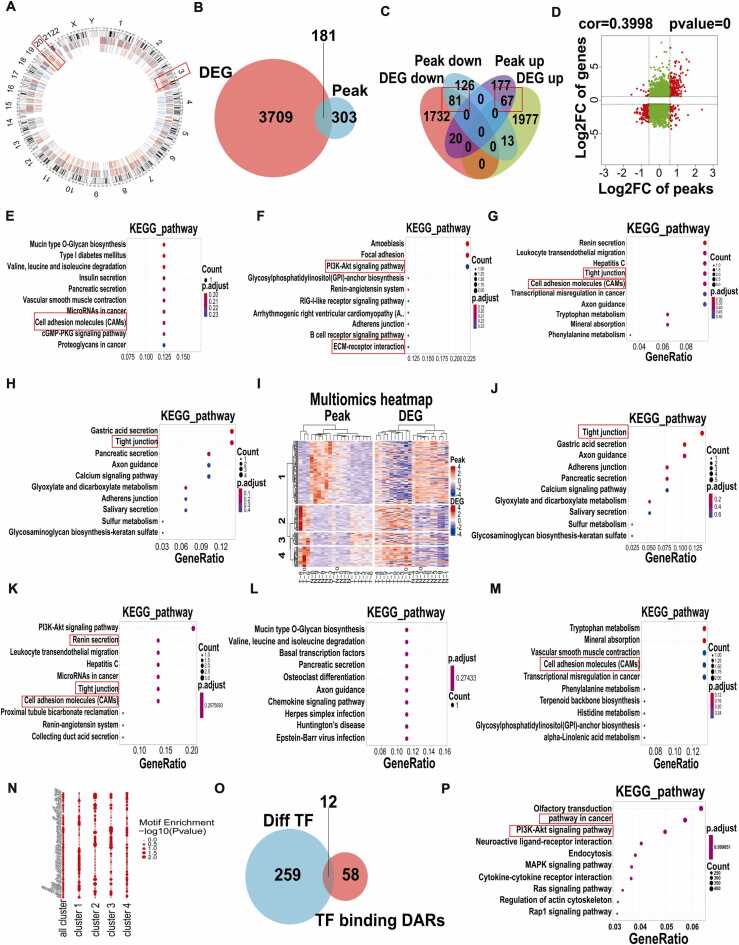
Performed reciprocal analysis of DARs and DEGs. (A-B) Circos plots of genome-wide differences in ATAC-seq and RNA-seq results. (C) Intersection analysis chart of DARs and DEGs up-regulated and down-regulated. (D) The correlation of the two omics as a whole is shown in the form of a nine-quadrant diagram. (E-H) KEGG enrichment analysis of different cluster intersection differential genes. (I) Heatmaps of overlapping DEGs from ATAC-seq data and transcription data. (J-M) KEGG enrichment analysis were performed for the screened overlapped mRNA. (N) Transcription factor enrichment analysis of each cluster. (O) Differential TF and TF binding to DARs intersection diagram. (P) KEGG enrichment analysis diagram of differential TF and TF binding to DARs.

The ATAC-seq technique predicts the binding of transcription factors (TFs), whereas RNA-seq reveals the transcription of TFs and can predict their potential target genes. Thus, a combined analysis of these two approaches can identify differentially expressed TFs that are simultaneously bound to differentially accessible regions (DARs). In other words, this analysis identifies TFs that are significantly differentially expressed and localized within DAR regions, which may play crucial roles in both chromatin accessibility and transcriptional regulation. The combined analysis of DARs and differentially expressed genes (DEGs) between normal (N) and tumor (T) groups revealed that 181 miRNAs were shared between the two sets of differential genes (Fig. 4A-B). Further analysis of the intersection between up-regulated and down-regulated DARs and DEGs showed that 81 DARs and DEGs overlapped in the down-regulated differential genes, while 67 overlapped in the up-regulated differential genes (Fig. 4C). The correlation between these genes across the two omics datasets was subsequently visualized in a nine-quadrant diagram (Fig. 4D).

KEGG enrichment analysis was performed on the differentially expressed genes from the cluster intersections to elucidate the primary functions of the four clusters (Fig. 4E-H). The intersection of differential genes from ATAC-seq and transcriptional data was visualized in a clustering heatmap (Fig. 4I). These overlapping differential genes were identified as potentially important regulators of gastric cancer development. KEGG enrichment analyses of these genes (Fig. 4J-M) were consistent with the results of single DARs enrichment analysis. Notably, tight junctions, cell adhesion molecules (CAMs), and the PI3K-Akt signaling pathway were identified as key mechanisms in gastric cancer progression. Additional KEGG and GO enrichment analyses were conducted for several distinct quadrants (Fig. S1). These analyses further suggested that, in addition to CAMs, extracellular matrix (ECM) receptors and signaling pathways regulating cell proliferation, such as p53 and the cell cycle, are also primarily involved in gastric cancer development. Tight junctions, CAMs, and the PI3K-Akt signaling pathway again emerged as critical mechanisms. Enrichment analysis of transcription factors in each cluster identified ZNF263 and PBX3 as key regulators, particularly involved in cluster 2 (Fig. 4N). Cluster 2 predominantly regulates tight junctions, CAMs, and the PI3K-Akt signaling pathway. The intersection of different TFs and TF binding to DARs revealed that 12 genes overlapped between the two groups (Fig. 4O). KEGG and GO enrichment analyses indicated that the PI3K-Akt pathway is a major driver of gastric cancer progression (Fig. 4P and Fig. S2). In summary, the comprehensive analysis of DARs and DEGs suggests that tight junctions, CAMs, and cell proliferation pathways, such as PI3K-Akt, p53, and the cell cycle, are critical signaling pathways in the development of gastric cancer.

### 3.5 High expression of FAM117A gene or low expression of PIGU gene can inhibit the proliferation of gastric cancer cell

**Fig. 5:**
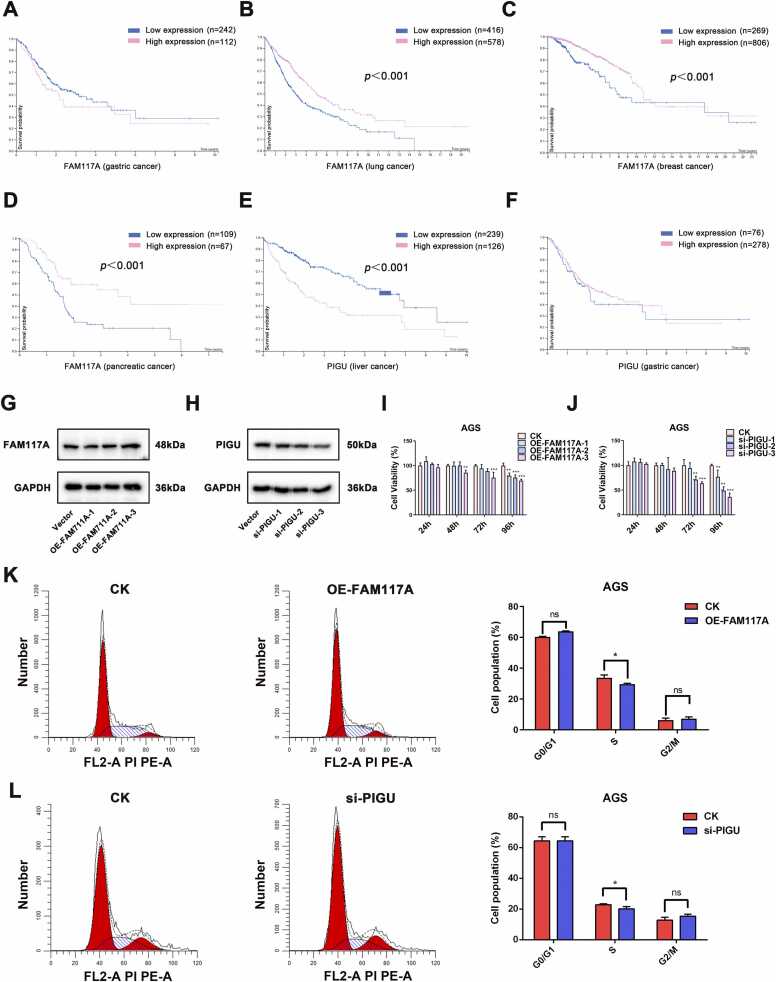
High expression of FAM117A gene or low expression of PIGU gene can inhibit the proliferation of gastric cancer cell. (A-C) The relationship between the level of FAM117A in GC, lung cancer and breast cancer and prognosis was investigated through https://www.proteinatlas.org/. (D-F) The relationship between the level of PIGU in pancreatic cancer, liver cancer and GC and prognosis was investigated through https://www.proteinatlas.org/. (G) Western blot analysis of FAM117A expression in AGS cells transfected with varying FAM117A concentrations. (H) Western blot analysis of FAM117A expression in AGS cells transfected with varying si-PIGU concentrations. (I) CCK-8 assay of AGS cell viability at 24–96 h post FAM117A transfection. (J) CCK-8 assay of AGS cell viability at 24–96 h post si-PIGU transfection. (K) Cell cycle distribution in FAM117A-transfected AGS cells by flow cytometry. (L) Cell cycle distribution in si-PIGU-transfected AGS cells by flow cytometry (*P＜0.1, **P＜0.01, ***P＜0.001. n = 3).

In gastric cancer, the higher expression of FAM117A is associated with a poorer prognosis, although the difference is not statistically significant (p > 0.05) (Fig. 5A). In contrast, lower expression of FAM117A correlates with worse prognosis in lung cancer (Fig. 5B) and breast cancer (Fig. 5C). However, in pancreatic cancer, a lower expression of PIGU is associated with a worse prognosis (Fig. 5D), whereas higher expression of PIGU in liver cancer is linked to poorer outcomes (Fig. 5E), a pattern that is also observed in gastric cancer (Fig. 5F). These findings were obtained through research using the Human Protein Atlas (https://www.proteinatlas.org/). Therefore, FAM117A and PIGU may play roles in regulating the occurrence and progression of gastric cancer. However, the specific regulatory mechanisms of FAM117A and PIGU remain unclear. To further investigate the effects of FAM117A overexpression and PIGU knockdown on AGS cells, we conducted preliminary experiments. Based on the results presented in Fig. 5G and H, transfection scheme 3 was selected as the intervention condition for subsequent experiments. To evaluate the inhibitory effects of upregulated FAM117A or downregulated PIGU on AGS cells in vitro, we performed CCK8 assays on OE-FAM117A and Si-PIGU AGS cells at various time points. As shown in Fig. 5I and J, overexpression of FAM117A and knockdown of PIGU significantly inhibited the proliferation of gastric cancer cells, with cell activity decreasing markedly over time. As expected, high levels of FAM117A and low levels of PIGU contributed to cell cycle arrest in AGS cells (Fig. 5K and L).

### 3.6 FAM117A regulates cell adhesion factors and PIGU regulates p53

**Fig. 6:**
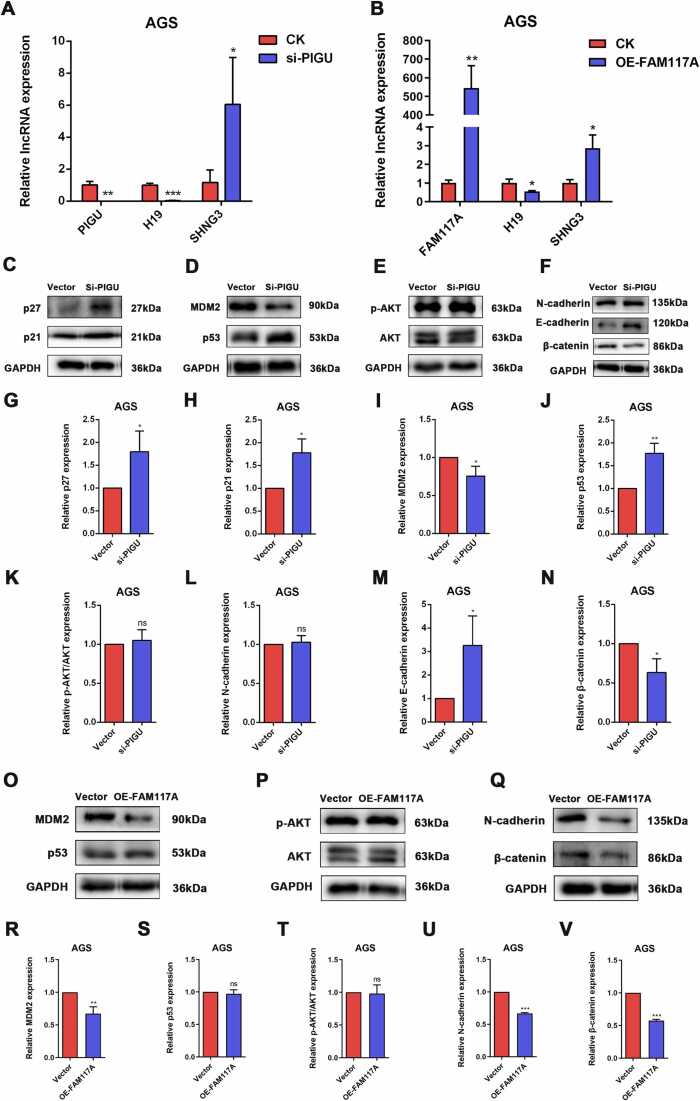
FAM117A regulates cell adhesion factors and PIGU regulates p53. (A) AGS cells were knock-down of PIGU, and the relative lncRNA expression of PIGU, H19 and SNHG3 were measured using RT-qPCR. (B) AGS cells were overexpression of FAM117A, and the relative lncRNA expression of FAM117A, H19 and SNHG3 were measured using RT-qPCR. (C-F) AGS cells were knock-down of PIGU, and the relative protein expression of p21, p27, MDM2, p53, E-cadherin, N-cadherin and β-catenin were measured using western blotting. The quantitative data were showed in (G-N). (O-Q) AGS cells were overexpression of FAM117A, and the relative protein expression of MDM2, p53, p-AKT, AKT, N-cadherin and β-catenin were measured using western blotting. The quantitative data were showed in (R-V). (*P＜0.1, **P＜0.01, ***P＜0.001. n = 3).

We further investigated the potential target genes or proteins influenced by FAM117A and PIGU in the development of gastric cancer cells. Long non-coding RNAs (lncRNAs) such as H19 and SNHG3 are crucial developmental regulators that play significant roles in tumorigenesis. Our results revealed that the knockdown of PIGU or the overexpression of FAM117A led to a downregulation of H19 in AGS cells compared to the control group (Fig. 6A and B). This suggests that H19 is a key downstream regulatory factor in the anti-tumor process involving high FAM117A expression and low PIGU expression. Western blotting results demonstrated that silencing PIGU (si-PIGU) increased the protein expression of p27, p21, p53, and E-cadherin, while inhibiting the expression of MDM2 and β-catenin in AGS cells (Fig. 6C-N). Notably, the expression of p-AKT/AKT and N-cadherin remained unaffected (Fig. 6C-N). Additionally, overexpression of FAM117A (OE-FAM117A) inhibited the protein expression of MDM2, N-cadherin, and β-catenin but had no significant effect on p53 and p-AKT/AKT expression (Fig. 6O-V). The above result has also been verified in MKN45 cells (Fig. 3S). These findings confirm that FAM117A and PIGU do not impact the PI3K/Akt signaling pathway but are capable of regulating the expression of cell adhesion factors and extracellular matrix (ECM)-related proteins. Moreover, they exert regulatory effects on p53 expression and the cell cycle.

## 4 Discussion

Gastric cancer (GC) is the fifth most common cancer and the third leading cause of cancer-related mortality worldwide [26]. GC is a complex, multifactorial disease. Its onset and progression typically follow a sequence, beginning with either inactive or chronic active gastritis, progressing to gastric precancerous lesions (GPLs), such as atrophic gastritis, intestinal metaplasia, and atypical hyperplasia, and ultimately culminating in the development of adenocarcinoma. This sequence is commonly referred to as the Correa cascade [27], [28]. Notably, the incidence of GC in patients with atrophic gastritis has been reported to be approximately 100 × 10⁻⁵ per year [29]. In patients with intestinal metaplasia (IM), the overall incidence rate of GC is 3.38 cases per 1000 person-years, with a higher incidence rate observed in those with incomplete IM (6.60 cases per 1000 person-years) [30]. Furthermore, in patients with low-grade dysplasia, the cumulative incidence of GC is approximately 3.1 %, while in those with high-grade dysplasia, the cumulative incidence increases to approximately 29.5 %. These findings suggest that not all GPLs inevitably progress to GC. The risk of progression is significantly higher in lesions exhibiting more severe pathology and greater histological irregularity. Currently, substantial progress has been made in research on therapeutic targets for GC and the identification of key regulatory transcription factors. Targeted therapies, such as VEGFR-2 inhibitors, are already available [31]. However, studies focusing on targets throughout the entire pathogenesis of GC remain limited, and understanding of molecular targets prior to the onset of GC, as well as in early-stage GC, remains insufficient. The GPL stage represents a critical juncture in the Correa cascade of gastric cancer, and the regression of GPLs is considered a secondary prevention strategy for GC [32], [33]. However, modern medicine currently lacks specific drugs and well-defined targets for treating GPLs. Therefore, identifying novel drugs that act on multiple steps of the Correa cascade and function as multi-phenotypic modulators is an important direction for future drug discovery.

In this study, we performed comprehensive transcriptome sequencing (mRNA, miRNA, lncRNA) and ATAC-seq analysis on samples from three distinct groups. Our results indicated that lncRNAs H19 and SNHG3 may serve as key regulated competing endogenous RNAs (ceRNAs). Through ATAC-seq analysis of differential accessible regions (DARs), we identified PBX3 and ZNF263 as potential transcription factors involved in regulation. Subsequent cellular-level validation of key differentially expressed genes (DEGs) revealed that FAM117A and PIGU exhibited highly consistent expression trends across cellular, transcriptomic, and database levels. These findings support the notion that tight junctions and cell adhesion molecules (CAMs) are critical pathways in gastric cancer progression at the transcription factor level. Further analysis explored the signaling pathways involved in gastric cancer development. Consistent with our transcription factor enrichment analysis, pathways such as PI3K/Akt, p53, and the cell cycle emerged as significant regulators of cell proliferation. This evidence underscores the importance of inter-cell adhesion, extracellular matrix (ECM) remodeling, and dysregulated proliferation in the gastric cancer continuum. As well-established in the literature, the PI3K/Akt and p53 signaling pathways play central roles in tumor regulation; however, targeted therapeutic options for gastric cancer within these pathways remain sparse, with drug development proving to be challenging. At the level of tight junction proteins, claudin-targeting drugs for gastric cancer have been introduced [34]. However, new treatments targeting CAMs and ECM are still lacking. CAMs are a group of molecules that mediate contact and binding between cells, or between cells and the ECM [35], [36]. Therefore, it is crucial to further explore the upstream regulatory factors governing cell proliferation and CAMs, as they represent potential avenues for therapeutic development in gastric cancer.

Among the previous findings, this study identified FAM117A and PIGU as crucial genes involved in the gastric cancer continuum, suggesting that dysregulation of cell proliferation and alterations in cell adhesion molecule (CAM) expression contribute significantly to gastric cancer development. Consequently, these two genes were selected for further experimental validation. Database analysis indicated that low FAM117A expression and high PIGU expression may promote gastric cancer progression. Subsequent experiments using a plasmid-based transfection system in gastric cancer cells demonstrated that overexpression of FAM117A and knockdown of PIGU inhibited cell proliferation, regulated the cell cycle, and induced cell cycle arrest, thereby confirming their role in the regulation of gastric cancer. RT-qPCR assays further revealed that overexpression of FAM117A and knockdown of PIGU suppressed H19 expression, without affecting SNHG3 expression. H19, a well-known lncRNA, has been implicated in gastric cancer promotion [37], [38]. These results suggest that FAM117A and PIGU may influence H19 expression to some extent. Subsequently, the effects of FAM117A and PIGU on the p53 and PI3K/Akt signaling pathways were explored. The results indicated that PIGU knockdown significantly modulated the p53 pathway, while FAM117A only affected MDM2 expression, without impacting p53 itself. Neither gene influenced the PI3K/Akt pathway. Finally, we investigated two classical cell adhesion molecules, cadherins, which are homophilic CAMs that depend on Ca²⁺ for function [39]. It is well established that over 200 types of epithelial-mesenchymal transition (EMT), extracellular matrix (ECM), and CAMs exist in humans. In gastric epithelial tissues, E-cadherin and N-cadherin are key targets regulating EMT, ECM, and CAM dynamics. It was found that FAM117B promotes the growth of gastric cancer by targeting the KEAP1/NRF2 signaling pathway. β-catenin has been shown to regulate EMT and cell proliferation [40]. This study found that overexpression of FAM117A could inhibit the expression of N-cadherin and β-catenin. It was proved that FAM117A mainly regulates the Wnt signaling pathway to regulate the transformation and malignant proliferation of gastric epithelial EMT. Recent studies found that PIGU can promote the progression of hepatocellular carcinoma by activating the NF-κB pathway and increasing immune escape [3]. In our study, PIGU knockdown inhibited N-cadherin and β-catenin expression while promoting E-cadherin expression, suggesting that PIGU is a significant upstream factor regulating the p53 signaling pathway and the cell cycle. Furthermore, PIGU appears to have a substantial regulatory impact on CAMs. Both FAM117A and PIGU are pivotal genes in the regulation of gastric cancer and represent important targets in the gastric cancer continuum.

In conclusion, this study conducted full transcriptome sequencing analysis and ATAC-seq analysis on human normal gastric mucosa tissues, gastric precancerous tissues and gastric cancer tissues, as well as verification of corresponding cell experiments, and proved that mutations in cell adhesion factors, such as epithelial transformation disorders such as EMT and abnormal cell proliferation, were the main causes of gastric cancer trilogy progression. This study further found that FAM117A and PIGU represent promising prognostic markers for assessing progression risk in gastric precancerous lesions.

## 5 Limitations

Our study has identified the potential regulatory roles of FAM117A and PIGU in gastric cancer development through multi-omics analysis and cellular experiments, but several limitations should be noted. First, although we observed the effects of FAM117A and PIGU on the p53 signaling pathway and cell adhesion molecules at the cellular level, we have not fully confirmed these regulatory mechanisms through in-depth molecular studies, such as ChIP-seq to verify transcription factor binding sites or luciferase reporter assays to validate interactions between lncRNAs and mRNAs. Second, while we identified potential associations between H19, SNHG3, and FAM117A/PIGU, these findings are currently based on correlative analyses and lack direct functional validation, such as RNA pull-down experiments to demonstrate their interactions. Lastly, our study is limited by a relatively small sample size and primarily relies on cellular experiments for validation, lacking in vivo model support. These limitations may affect the comprehensiveness and generalizability of our conclusions. Future research will further validate these findings in larger cohorts and in vivo models and conduct additional functional and mechanistic studies to elucidate the specific roles of FAM117A and PIGU in gastric carcinogenesis.

## Author contributions

JT, YW and JZ conceived and directed the study. NC, YW, QL, JD analyzed the data, interpreted the results, and wrote the manuscript. DY, MZ and CG processed the data and conducted bioinformatics analysis. TW and JM collected all patient samples and helped with the whole-transcriptome sequencing and RT-PCR experiments. All authors read and approved the manuscript.

## CRediT authorship contribution statement

**Cui Guo:** Data curation. **Tingyao Wang:** Investigation. **Dan Yao:** Investigation. **Maoyuan Zhao:** Formal analysis. **Yumei Wang:** Conceptualization. **Jinhao Zeng:** Funding acquisition, Conceptualization. **Jia Ma:** Resources. **Jianyuan Tang:** Funding acquisition. **Nianzhi Chen:** Writing – original draft. **Yueqiang Wen:** Writing – review & editing. **Qingsong Liu:** Methodology. **Jing Du:** Data curation.

## Ethics approval and consent to participate

There are no direct clinical activities associated with the study and only uses primary human samples for analysis, and this study was approved by the Ethics Committee of the Hospital of Chengdu University of Traditional Chinese Medicine (approval no. 2018KL‑023), and informed consent was obtained from all participants.

## Funding

This work was supported by the 10.13039/501100001809National Natural Science Foundation of China (grant no. 82174346), the Program of Science and Technology Department of Sichuan Province (Grant No. 2023NSFSC0039), Xinglin Scholar Research Promotion Project of Chengdu University of TCM (Grant No. QJJJ2024005), the Joint Innovation Fund of Health Commission of Chengdu and 10.13039/501100008402Chengdu University of Traditional Chinese Medicine (grant nos. LH202402044 and WXLH202403008), the "First-class Discipline Peak Matrix Key Research 'Top-down Leadership' Project (Traditional Chinese Medicine Regulatory Science)" (Gant No. 2025ZD008).

## Declaration of Competing Interest

The authors declare that they have no known competing financial interests or personal relationships that could have appeared to influence the work reported in this paper.

## Data Availability

In this study, the public data used and analyzed are summarized in “Methods”. All the source code used in this article to analyze data and generate the results and figures can be obtained on GitHub by visit https://github.com/lqscptp/ceRNA.git.

## Appendix A Supplementary material

Figure S1 KEGG and GO analysis for the nine quadrants. (A-F) KEGG and GO analysis plots for the difference quadrants

Figure S2 GO enrichment analysis of diff-TF binding to DARs. (A-C) GO enrichment analysis diagram of differential TF and TF binding to DARs

Figure S3 FAM117A and PIGU regulates cell adhesion in MKN45. (A-B) MKN-45 cell were knock-down of PIGU, and the protein expression of MDM2 were measured using western blotting. (C-F) MKN-45 cells were overexpression of FAM117A, and the relative protein expression of MDM2, N-cadherin and β-catenin were measured using western blotting. The quantitative data were showed in (D-F). (**P*＜0.1, ***P*＜0.01, ****P*＜0.001. n = 3)
