# Supplementary figures and images for: FAM117A and PIGU regulate the trilogy of gastric carcinogenesis
Source: Comput Struct Biotechnol J. 2025 Sep 7;27:3952–65. doi: 10.1016/j.csbj.2025.08.036 (PMC12799953; doi:10.1016/j.csbj.2025.08.036)

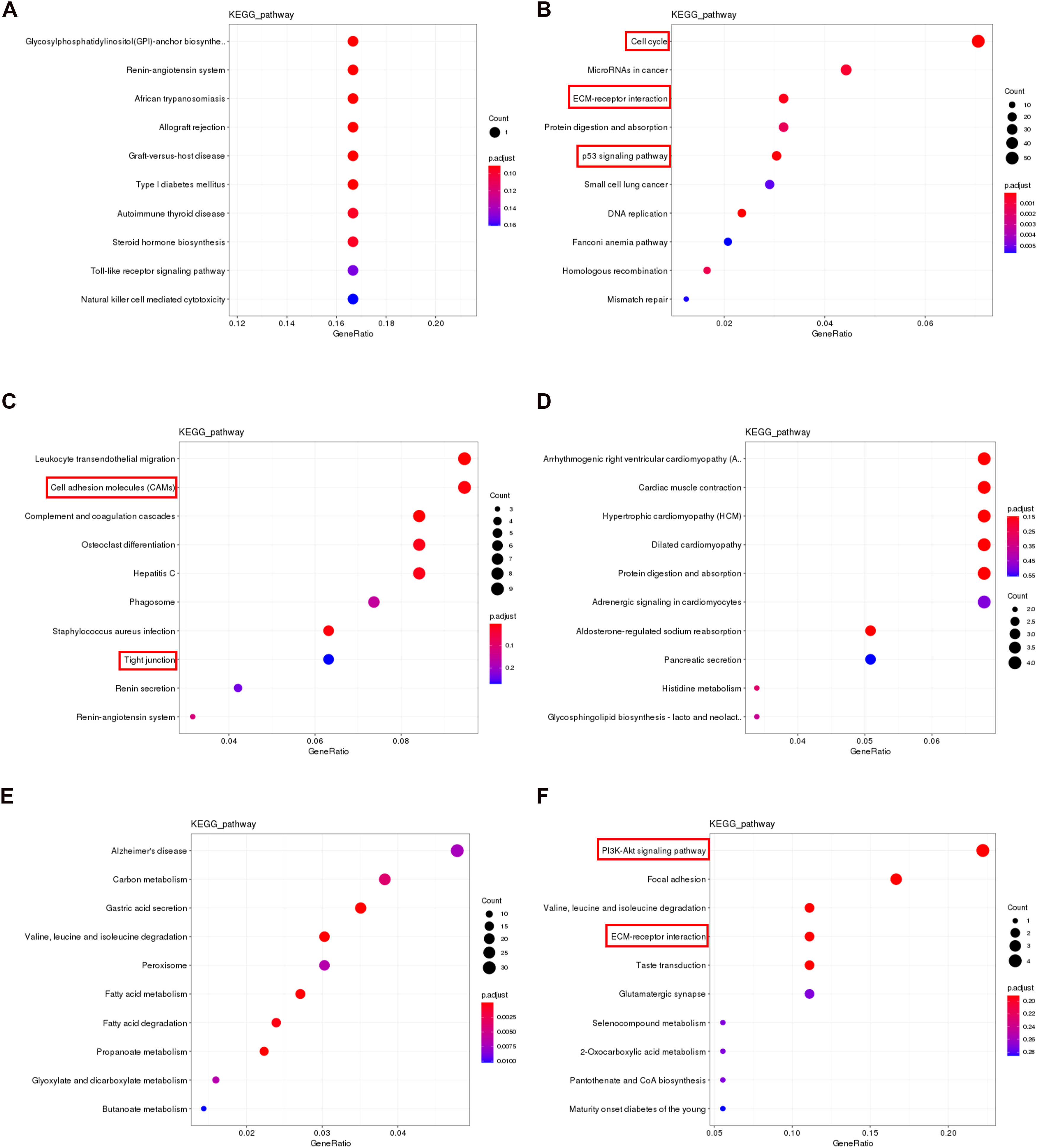

Supplement: Figure S1 — KEGG and GO analysis for the nine quadrants. (A-F) KEGG and GO analysis plots for the difference quadrants [file mmc1.jpg]

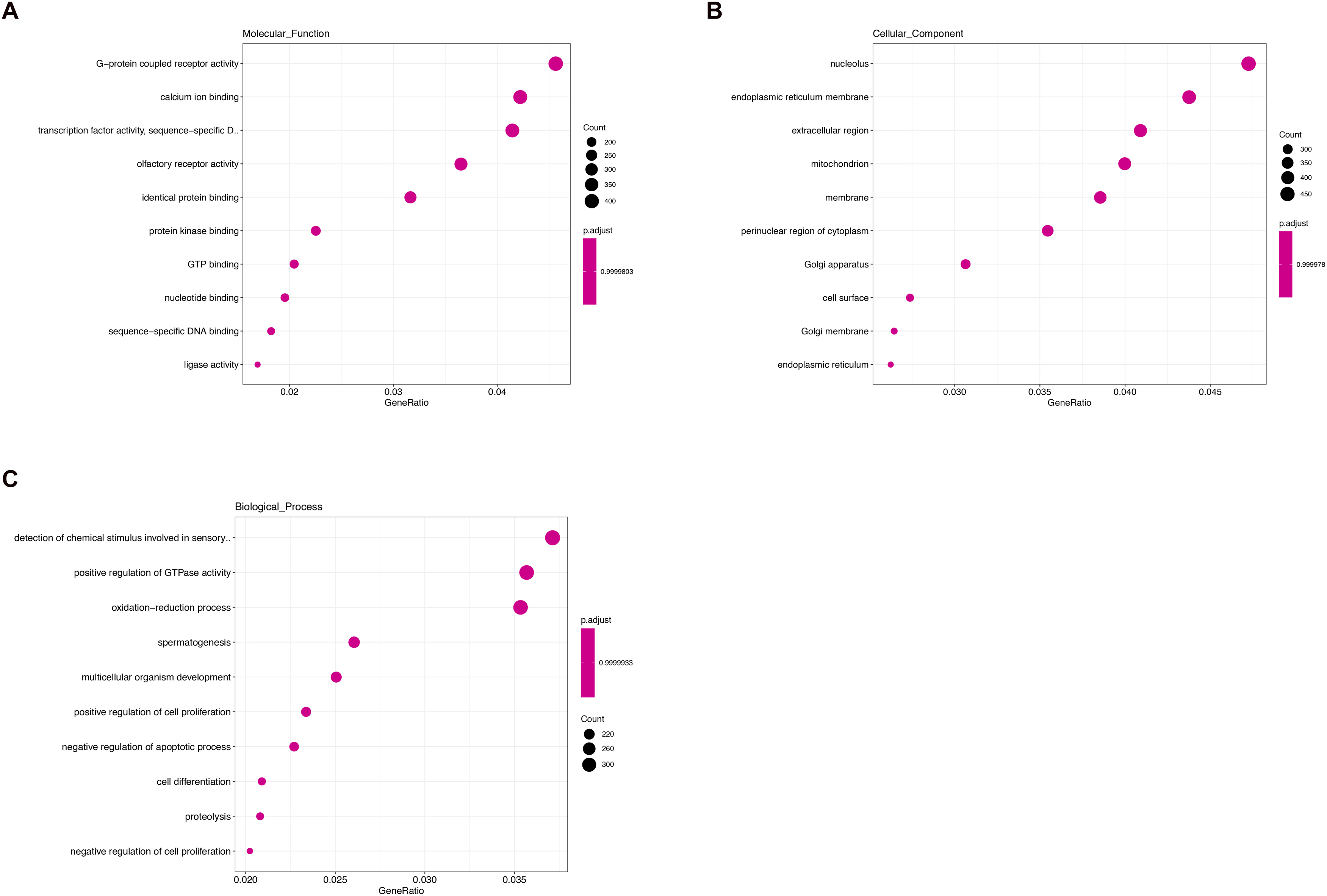

Supplement: Figure S2 — GO enrichment analysis of diff-TF binding to DARs. (A-C) GO enrichment analysis diagram of differential TF and TF binding to DARs [file mmc2.jpg]

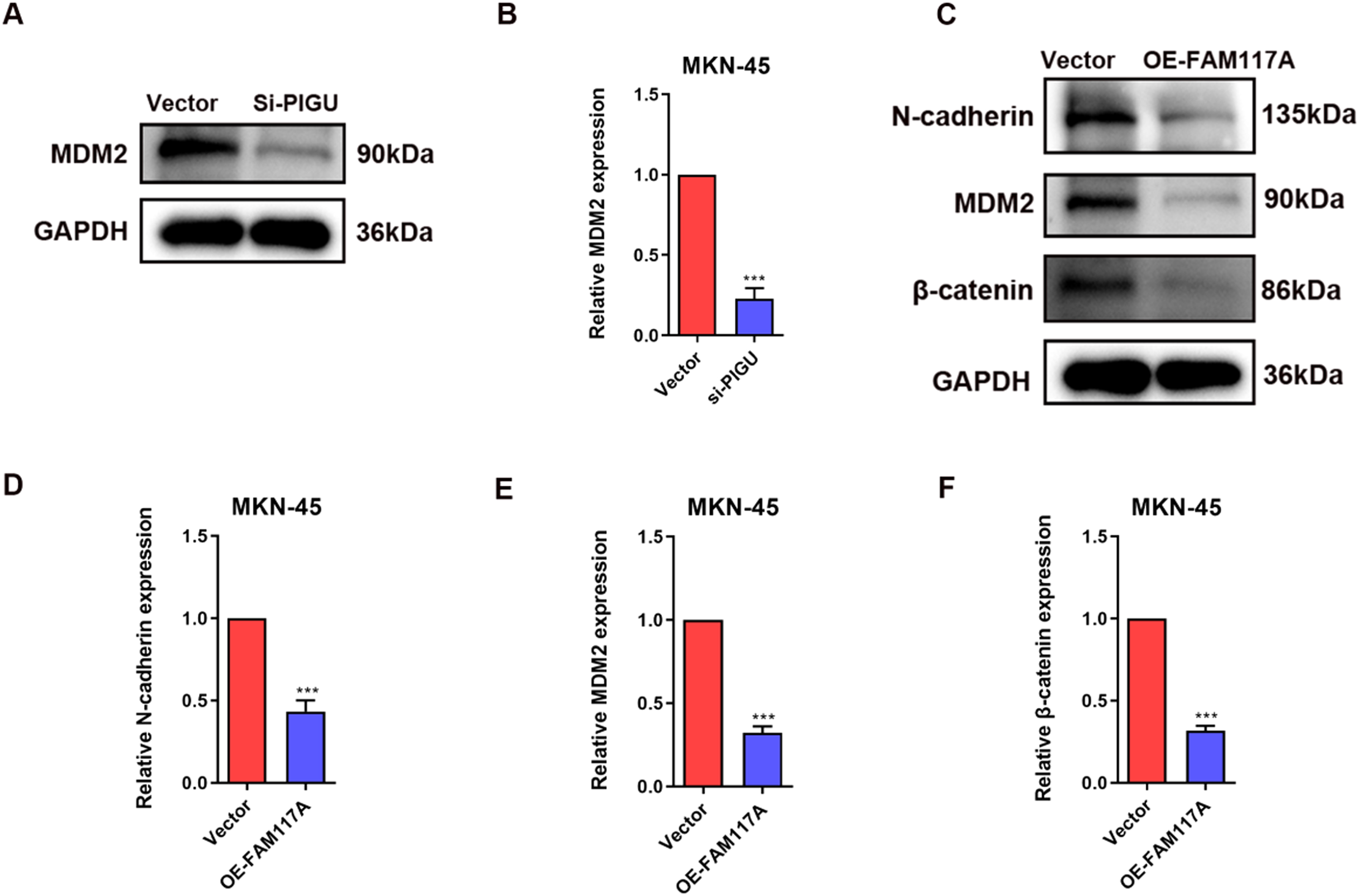

Supplement: Figure S3 — FAM117A and PIGU regulates cell adhesion in MKN45. (A-B) MKN-45 cell were knock-down of PIGU, and the protein expression of MDM2 were measured using western blotting. (C-F) MKN-45 cells were overexpression of FAM117A, and the relative protein expression of MDM2, N-cadherin and β-catenin were measured using western blotting. The quantitative data were showed in (D-F). (*P＜0.1, **P＜0.01, ***P＜0.001. n = 3) [file mmc3.jpg]
